# Vital Statistics

**Published:** 1883-01

**Authors:** 


### Vital Statistics.

The fiscal year of the Board of Health closed at noon Jan. 1, and it was then ascertained that during the period mentioned 37,951 deaths, 27,351 births and 11,085 marriages had been recorded, which was a decrease of 673 deaths and an increase of 1,221 births and 1,008 marriages over the figures of the previous year, and an increase of 9,609 deaths, 1,748 births and 2,639 marriages over those of 1880. The following table shows the statistics by months and the totals for the two previous years:

| Month. | Deaths. | Births. | Marriages. | Month. | Deaths. | Births. | Marriages. |
|---|---|---|---|---|---|---|---|
| January | 3,498 | 2,278 | 925 | September | 2,579 | 2,365 | 927 |
| February | 3,298 | 2,092 | 946 | October | 2,588 | 2,471 | 1,064 |
| March | 3,481 | 2,506 | 820 | November | 2,461 | 2,292 | 1,000 |
| April | 3,395 | 2,042 | 784 | December | 2,616 | 2,531 | 1,069 |
| May | 3,353 | 2,150 | 1,000 | | | | |
| June | 2,871 | 2,065 | 980 | Totals | 37,951 | 27,321 | 11,085 |
| July | 4,482 | 2,060 | 732 | Totals, 1881 | 38,624 | 26,130 | 10,077 |
| August | 3,329 | 2,469 | 838 | Totals, 1880 | 28,342 | 25,573 | 8,446 |

*The Varieties of Disease.*—Of the deaths during the year 1,389 were due to violence, 199 to suicide, 198 to drowning, 103 to solar heat, 269 to smallpox, 912 to measles, 2,070 to scarlet fever, 1,521 to diphtheria, 730 to croup, 655 to whooping cough, 151 to erysipelas, 66 to typhus fever, 363 to typhoid fever, 533 to malarial fever, 408 to puerperal diseases, 237 to cerebro-spinal meningitis, 232 to alcoholism, 184 to rheumatism and gout, 625 to cancer, 5,245 to consumption, 1,591 to bronchitis, 3,408 to pneumonia, 1,443 to heart disease, 937 to marasmus and scrofula, 652 to hydrocephalus, 745 to meningitis and eucephalitis, 640 to convulsions, 609 to apoplexy, 1,860 to Bright's disease, 2,980 to diseases of brain and nerves, 943 to gastritis and peritonitis and 4,058 to diarrhœal diseases ; 12,522 belonged to the zymotic class, 7,844 were constitutional, 15,000 were local, and 2,196 were developmental. Of those that died 9,875 were children under 1 year, 13,447 were under 2 years, 17,305 were under 5 years, and 2,342 were over 70 years ; 8,311 were married and 3,903 widowed ; 7,518 of the deaths in public institutions, 20,690 in tenements, 8,832 in houses containing families of three and less and 388 in hotels and boarding-houses ; 456 dead bodies were found in the rivers and streets ; 239 of the pauper dead were delivered to the surgeons for dissection.

*Accidental Deaths and Suicides.*—Twenty-five persons were accidentally poisoned—1 by taking rat poison, 1 by soothing syrup, 1 by acetic acid, 4 by opium, 7 by lead, 2 by laudanum, 1 by chlorate of potash, 2 by iodine, 1 by carbolic acid, 1 by whiskey, 1 by bromide of potash, 1 by morphine, 1 by hydrate of chloral and 1 by arsenic. One hundred and fifty-eight of the accidental deaths were caused by fractures, 31 by wounds, 110 by being run over, 356 by falls, 53 by scalds and 82 by burns. There were 75 homicides during the year, 34 resulting from blows, 13 from cuts and stabs, 19 from shooting, 1 from being thrown from a roof, 1 from being thrown from a window, 1 from elevated train, 1 from scalds, 3 from miscarriage, and 2 were asphyxiated. One hundred and sixty-five males and 34 females committed suicide; 1 was a boy of 13, another a girl of 14, and 2 were over 80 years old; 14 killed themselves by drowning, 29 by hanging, 20 by cutting, 53 by shooting, 12 by jumping out of windows, 2 by throwing themselves in front of locomotives, 18 by taking Paris green, 4 by laudanum, 8 by arsenic, 4 by prussic acid, 5 by oxalic acid, 2 by strychnine, 3 by carbolic acid, 7 by rat poison, 1 by aconite, 1 by inhaling chloroform, 1 by unknown irritant poison, and 1 was asphyxiated by the fumes of charcoal; 47 were unmarried, 79 married and 24 widowed; 3,879 inquests were held by the Board of Coroners.

*Deaths of Centenarians.*—Thirteen persons died during the year whose ages were 100 years and over. They were as follows:

John Davis (colored), single, born in this city, aged 104 years; Margaret Martin (colored), widow, born in this city, aged 103 years; Dennis Skelly, widower, born in Ireland, aged 105 years; Ann Spencer, widow, born in Ireland, aged 105 years; Ann Donnelly, widow, born in Ireland, aged 100 years; Catharine Stevenson, widow, born in Ireland, aged 100 years; Mary Smith (colored), widow, born in Virginia, aged 107 years; Bridget Haley, widow, born in Ireland, aged 107 years; Sarah A. Saunders (Indian), widow, born in Pennsylvania, aged 100 years; Mary Gillen, widow, born in Ireland, aged 102 years; James Gilman, widower, born in Ireland, aged 100 years; Elizabeth Rice, widow, born in Ireland, aged 100 years, and Margaret Londrigan, widow, born in Ireland, aged 100 years.

There were 36 persons whose ages reached 90 years and over; 14 of them were men and 22 were women; 9 were natives of the United States, 18 of Ireland, 6 of Germany, 1 was a native of Poland, 1 of Bohemia and 1 of France.

The fathers of 18,158 of the children born were of foreign birth and of 9,163 natives, and the mothers of 15,960 of the children were of foreign birth and of 11,361 natives; 26,999 of the children born were white and 329 colored; 14,068 were males and 13,255 females. There were 187 twin births and one mother was delivered of triplets.

*Marriage Statistics.*—During the year 11,085 marriages were performed, and of the grooms 10,842 were white and 243 colored, and of the brides 10,852 white and 233 colored; 9,163 grooms were married for the first time, 1,455 had been previously married, 116 were married twice and 6 three times; 9,394 brides were married for the first time, 1,235 were widows, 46 had been married twice, and 1 was married to her fourth husband; 204 grooms and 2,651 brides were under 20 years, 14 grooms and 2 brides were between 65 and 70 years; 14 grooms were between 70 and 80 years, and 1 was between 80 and 90 years.

CONTAGIOUS DISEASES BY MONTHS.

| MONTH. | Scarlet Fever. | Diphtheria. | Typhoid Fever. | Typhus Fever. | Cerebro-spinal Meningitis | Measles. | Smallpox. |
|---|---|---|---|---|---|---|---|
| January | 1,329 | 687 | 46 | — | 24 | 866 | 187 |
| February | 1,167 | 461 | 30 | 16 | 23 | 825 | 183 |
| March | 1,054 | 451 | 25 | 38 | 20 | 756 | 143 |
| April | 888 | 391 | 22 | 45 | 17 | 637 | 69 |
| May | 758 | 323 | 25 | 57 | 23 | 768 | 71 |
| June | 367 | 229 | 20 | 42 | 31 | 299 | 37 |
| July | 154 | 172 | 40 | 4 | 16 | 130 | 9 |
| August | 115 | 141 | 96 | 4 | 20 | 56 | 1 |
| September | 87 | 116 | 117 | 1 | 20 | 21 | 2 |
| October | 110 | 153 | 147 | — | 12 | 40 | 3 |
| November | 124 | 185 | 68 | — | 12 | 60 | 3 |
| December | 199 | 190 | 48 | — | 16 | 164 | — |
| Totals | 6,793 | 3,504 | 684 | 207 | 234 | 4,633 | 708 |
| Totals for 1881 | 7,338 | 5,272 | 965 | 568 | 556 | 3,116 | 1,342 |
| Totals for 1880 | 3,048 | 3,307 | 508 | 2 | 197 | 3,891 | 67 |
| Totals for 1879 | 5,446 | 1,783 | 432 | 8 | 119 | 2,333 | 65 |

